# Potential biomarkers for the early detection of bone metastases

**DOI:** 10.3389/fonc.2023.1188357

**Published:** 2023-06-19

**Authors:** Yang Hao, Feifan Zhang, Yan Ma, Yage Luo, Yongyong Zhang, Ning Yang, Man Liu, Hongjian Liu, Jitian Li

**Affiliations:** ^1^ Laboratory of Molecular Biology, Henan Luoyang Orthopedic Hospital (Henan Provincial Orthopedic Hospital), Zhengzhou, China; ^2^ Henan University of Chinese Medicine, Zhengzhou, China; ^3^ Hunan University of Chinese Medicine, Changsha, China; ^4^ Department of Orthopaedics, the First Affiliated Hospital of Zhengzhou University, Zhengzhou, China

**Keywords:** bone metastases, biomarkers, ncRNAs, circulating tumor cells, exosome

## Abstract

The clinical manifestations of bone metastases are diversified while many sites remain asymptomatic at early stage. As the early diagnosis method is not perfect and the early symptoms of tumor bone metastasis are not typical, bone metastasis is not easy to be detected. Therefore, the search for bone metastasis-related markers is effective for timely detection of tumor bone metastases and the development of drugs to inhibit bone metastases. As a result, bone metastases can only be diagnosed when symptoms are found, increasing the risk of developing skeletal-related event (SREs), which significantly impairs the patient’s quality of life. Therefore, the early diagnosis of bone metastases is of great importance for the treatment and prognosis of cancer patients. Changes of bone metabolism indexes appear earlier in bone metastases, but the traditional biochemical indexes of bone metabolism lack of specificity and could be interfered by many factors, which limits their application in the study of bone metastases. Some new biomarkers of bone metastases have good diagnostic value, such as proteins, ncRNAs, circulating tumor cells (CTCs). Therefore, this study mainly reviewed the initial diagnostic biomarkers of bone metastases which were expected to provide references for the early detection of bone metastases.

## 1 Introduction

Bone metastasis occurs when tumor cells spread to the bones. When people suffering from cancer, with the progession of the disease, the cancer cells invade the blood vessels. As the blood flows, the cancer cells may travel to the bone marrow and continue to rise, forming bone metastases (1). Distant metastases are a typical characteristic of malignant tumor, as well as one of the main reasons leading to treatment failure of tumor patients (2). On average, 1 out of every 5 patients will suffer from bone metastases. Theoretically, almost all types of cancers may metastasize to bone, among which lung cancer, breast cancer and prostate cancer are the most frequent (3). Digestive tract tumors such as stomach cancer, bowel cancer, pancreatic cancer, etc., can also appear, relatively low risk. There are three types of bone metastases: osteolytic, osteoblastic and mixed (4, 5). Only clear diagnosis and symptomatic treatment will have beneficial clinical effect (6). Osteogenic bone metastases are widespread in prostate cancer, accounting for about 10% of bone metastases. Lytic bone metastases account for 70%, which are atypical lung and breast cancer (4).

The early diagnosis of malignant tumors is very critical to the recovery. In clinical practice, some of cancer patients showed symptoms such as waist and leg pain or anemia (especially those who had a history of this, such as rheumatic inflammation, lumbar disc herniation, etc.), but they did not pay enough attention (7). In fact, it is highly likely that this is a precursor of tumor bone metastases. If the bone lesions and complications of bone metastases cannot be treated reasonably, it will do great harm, such as pathological fractures, which often paralyze patients in bed, as well as the severe pain will seriously affect the quality of life of patients (8, 9).

Early diagnosis of bone metastases is of major importance. The main symptom of bone metastases is persistent pain with continuously aggravated, which may also cause mobility impairment. The commonly used imaging methods for the diagnosis of bone metastases have different characteristics. As for X-ray, specificity is high but sensitivity is low. The positive rate of bone ECT imaging is high, but there exist false positive and false negative problems (5, 10). CT and MRI have high specificity and accuracy, but are not appropriate for general examination. positron emission computed tomography PET has a high positive rate, but it doesn’t applicable to simple bone lesions, and the price is relatively high, which limited its application in clinic (11, 12). Theoretically, the changes of biochemical indexes of bone metabolism during bone metastases are earlier than those in imaging (13, 14). However, traditional biochemical indexes of bone metabolism with low specificity limits their application in the study of bone metastases (15, 16).

**Figure 1:**
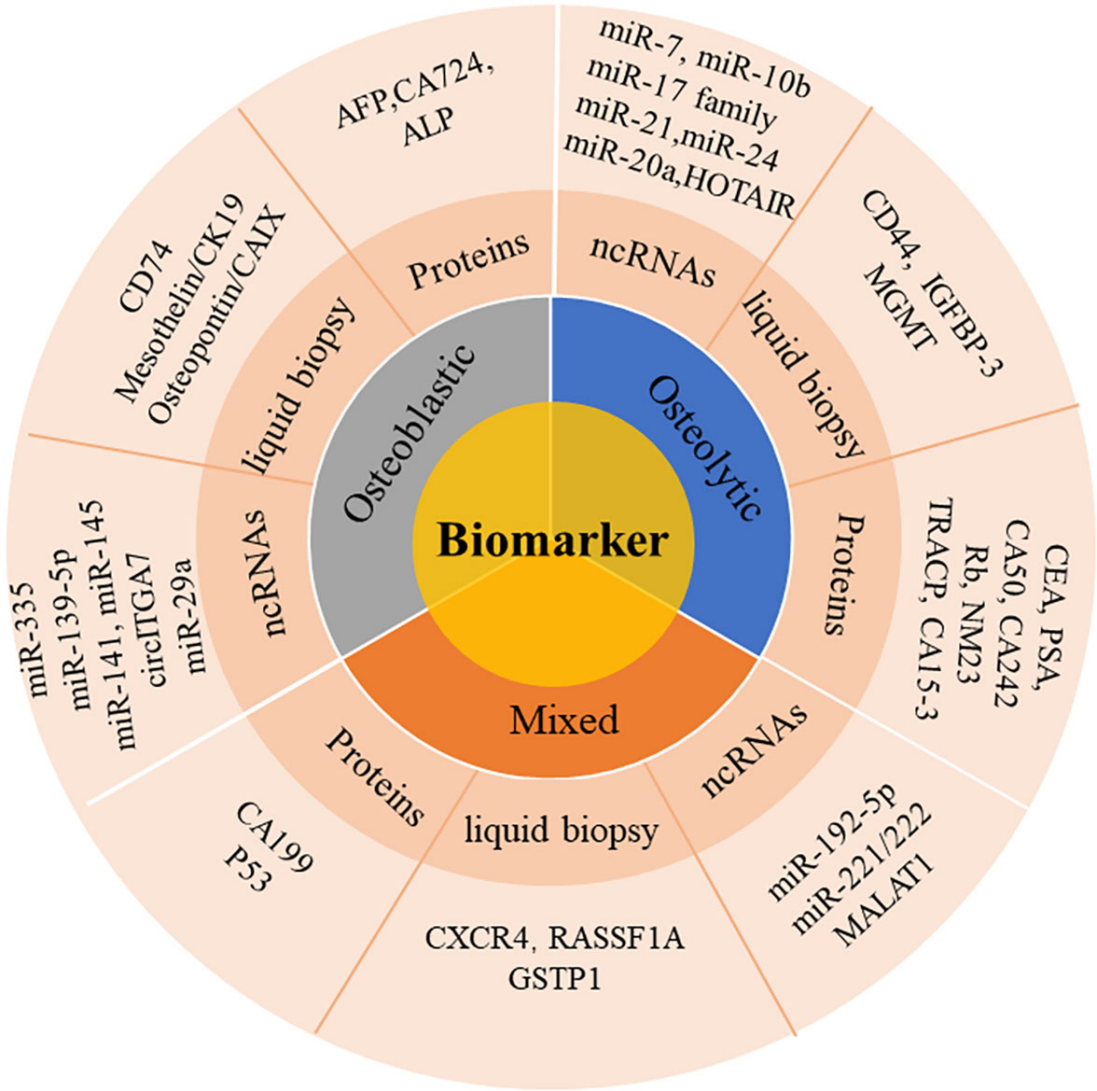
The role of partial biomarkers in bone metastase.

Some new biomarkers of bone metastases have good diagnostic value, such as proteins, ncRNAs, biomarkers in liquid biopsy and other biochemical indicators. These new types of biomarkers have demonstrated great potential in the initial diagnosis of bone metastases. In the study we searched relevant researches for bone metastases biomarkers, which mainly provides reference for early diagnosis of bone metastases, as shown in **Figure 1**.

## 2 Application of commonly used protein biomarkers in bone metastases

Protein biomarkers are most commonly used in the clinical diagnosis and prognosis of bone metastases. It indicates proteins in the blood whose presence or abnormal expression is often associated with certain types of tumors. These proteins can be detected in tumor cells, surrounding tissues, and blood, these biomarkers can be employed to monitor patient responsiveness and effectiveness during treatment. However, it is important to emphasize that a single blood biomarker is not enough to detect the tumor. It is usually used in conjunction with other tests, imaging and clinical symptoms to determine the status of the tumor. The presence of digestive system tumors and the occurrence of bone metastases may lead to increasing carbohydrate resistance, such as the indexes of alpha-fetoprotein (AFP), carcinoembryonic antigen (CEA), prostate specific antigen (PSA), CA199, CA724, CA50, and CA242.

Except that most commonly used for bone metastases tumor biomarkers include bone specific alkaline phosphatase (BALP), tartrate-resistant acid phosphatase (TRACP), tumor necrosis factor (TNF), carbohydrate antigen 15-3 (CA15-3). The exact contents were shown in **Table 1**. The diagnostic performance of each biomarker was presented shown in **Table 2**.

**Table 1:** Application of commonly used biomarkers of bone metastases.

Biobiomarker	Bone transition stage	Clinical application	Deregulation
AFP	Osteoblastic	Detect the occurrence and development of bone metastases, especially in breast cancer, lung cancer and colon cancer.	Low-expression
CEA	Osteolytic	Provide objective guidance for clinical treatment planning and treatment.	Over-expression
PSA	Osteolytic	Screening, diagnosis and efficacy evaluation of prostate cancer.	Over-expression
CA199	Mixed	Predict the malignant transformation and prognosis of liver cancer.	Over-expression
CA724	Osteoblastic	Clinical diagnosis and prognosis of found guilty of an important tumor biomarker in breast cancer.	Over-expression
CA50	Osteolytic	Detect bone metastases of liver cancer.	Low-expression
CA242	Osteolytic	An epigenetic specific antigen used to detect bone metastases in gastric cancer	Over-expression
Rb	Osteolytic	Rb plays an important role in the regulation of bone metastases suppressor genes such as Osteoprotegerin.	Low-expression
P53	Mixed	Patients with bone metastases expressing p53 have a poor prognosis.	Over-expression
NM23	Osteolytic	NM23 is associated with cell proliferation, invasion, and metastases of bone metastases, and is generally associated with poor treatment response and prognosis.	Low-expression
ALP	Osteoblastic	Reflecting bone metastases lesions, and is regarded as a biomarker of early differentiation of osteoblast precursor cells.	Over-expression
BALP	Osteolytic	BALP level is a key predictor of treatment response and prognosis of bone metastases.	Over-expression
TRACP	Osteolytic	A decline in TRACP levels is usually associated with a better prognosis for treatment. In addition, monitoring TRACP levels can also help determine the timing and regimen of treatment and possible problems with bone metabolism.	Over-expression
CA15-3	Osteolytic	CA15-3 levels are often elevated in breast cancer patients with bone metastases.	Over-expression

**Table 2:** Diagnostic performance of commonly used biomarkers of bone metastases in single study.

biomarkers	primary cancer types	Study population characteristics		Diagnostic performance				Ref.
		cases group	Controls group	Research method	Se. (%)	Sp. (%)	AUC	
AFP/AFP-L3	HCC	50	484	uTASWako i30	63.30	90.00	/	Tayob N et al. (2022) (17)
AFP	HCC	79	77	Microchip capillary electrophoresis	68.35	81.82	0.683-0.818	Park SJ et al. (18)
AFP	HCC	104	336	Retrospective analysis	71.00	91.00	/	Zhu AX et al. (19)
AFP	HCC	36	31	LC-MS	88.90	82.90	0.892	Luo et al. (20)
AFP	HCC	135	302	Genome-wide discovery	71.00	90.00	0.92	Chalasani NP et al. (21)
AFP	HCC	90	60	Immunohistochemical	82.60	96.20	/	Chen D et al. (22)
AFP	GCT	41	35	Retrospective analysis	71.00	80.00	/	Calaminus G et al. (23)
ALP	RCC	111	261	Histopathologic analysis	57.90	83.50	0.749	Chen XY et al. (24)
Calcium	RCC	111	261	Histopathologic analysis	36.80	95.20	0.633	Chen XY et al. (24)
HB	RCC	111	261	Histopathologic analysis	71.10	65.30	0.665	Chen XY et al. (24)
HB+ALP	RCC	111	261	Histopathologic analysis	47.40	91.00	/	Chen XY et al. (24)
HB+CA	RCC	111	261	Histopathologic analysis	34.20	97.60	/	Chen XY et al. (24)
ALP+CA	RCC	111	261	Histopathologic analysis	28.90	97.90	/	Chen XY et al. (24)
HB+CA+ALP	RCC	111	261	Histopathologic analysis	28.90	98.20	/	Chen XY et al. (24)
uNTX	NSCLC	100	50	Osteomark, Princeton, NJ	48.00	86.00	0.74	Tamiya et al. (25)
sNTX	NSCLC	100	50	Osteomark, Princeton, NJ	40.00	87.00	0.71	Tamiya et al. (25)
CTX	NSCLC	16	18	ELISA, RIA	73.70	86.70	0.68	Lumachi et al. (26)
ICTP	LC	47	44	Radioimmunoassay, immunoassay	71.40	87.90	/	Aruga et al. (27)
fDPD	LC	47	44	Radioimmunoassay, immunoassay	61.00	93.00	/	Aruga et al. (27)
PICP	LC	47	44	Radioimmunoassay, immunoassay	28.60	87.90	/	Aruga et al. (27)
BGP	LC	47	44	Radioimmunoassay, immunoassay	12.30	81.80	/	Aruga et al. (27)
ALP	LC	47	44	Radioimmunoassay, immunoassay	55.60	79.50	/	Aruga et al. (27)
BALP	LC	47	44	Radioimmunoassay, immunoassay	44.40	93.20	/	Aruga et al. (27)
ICTP	LC	140	50	Double-antibody Radioimmunoassay	92.00	70.00	0.816	Horiguchi et al. (28)
CEA	LC	140	50	Double-antibody Radioimmunoassay	60.00	55.00	0.571	Horiguchi et al. (28)
CYFRA 21-1	LC	140	50	Double-antibody Radioimmunoassay	60.00	45.00	0.538	Horiguchi et al. (28)
ProGRP	LC	140	50	Double-antibody Radioimmunoassay	42.00	65.00	0.557	Horiguchi et al. (28)
ALP	LC	140	50	Double-antibody Radioimmunoassay	22.50	92.00	0.654	Horiguchi et al. (28)
Ca	LC	140	50	Double-antibody Radioimmunoassay	0.070	100.00	0.321	Horiguchi et al. (28)
ALP	LC	30	152	Hitachi747 autoanalyzer	26.70	97.30	0.857	Min et al. (29)
ICTP	LC	130	135	ELISA	63.10	90.40	0.835	Tang et al. (30)
BAP	LC	130	135	ELISA	63.10	77.00	0.760	Tang et al. (30)
TRACP 5b	LC	130	135	ELISA	58.50	80.70	0.753	Tang et al. (30)
CTX	NSCLC	16	18	Automated Immunometric assay	73.30	86.70	0.794	Lumachi et al. (26)
CEA	NSCLC	16	18	ELISA	55.50	62.50	0.588	Lumachi et al. (26)
CYFRA	NSCLC	16	18	Immunochemiluminescent assay	65.00	78.60	0.706	Lumachi et al. (26)
TRAP5b	NSCLC	16	18	ELISA	30.40	76.20	0.676	Lumachi et al. (26)
PINP	NSCLC	16	18	RIA	72.20	81.20	0.765	Lumachi et al. (26)
ICTP	LC	21	65	ELISA	86.40	84.60	0.87	Yokoyama et al. (31)
TRACP5b	NSCLC	72	69	Immunoassay	63.90	76.80	0.749	Yao et al. (32)
PSA	PC	771	13	ELISA-PSA	91.30	98.70	/	Modoni et al. (33)
BSP	PC	42	41	ELISA	80.95	72.80	/	Wei et al. (34)
PSA	PC	42	41	ELISA	57.14	64.80	/	Wei et al. (34)
ICTP	PC	42	41	ELISA	69.05	76.80	/	Wei et al. (34)
ALP	PC	42	41	ELISA	71.43	88.80	/	Wei et al. (34)
PSA	PC	87	99	ELISA	46.77	53.33	/	Szot et al. (35)
PICP	BC	92	53	ELISA	28.10	83.90		Zissimopoulos et al. (36)
ICTP	BC	92	53	ELISA	48.60	94.00		Zissimopoulos et al. (36)
CEA	BC	92	53	ELISA	42.00	65.00		Zissimopoulos et al. (36)
CA15-3	BC	92	53	ELISA	78.00	86.00		Zissimopoulos et al. (36)
ICTP+CEA+CA15-3	BC	92	53	ELISA	82.00	96.00		Zissimopoulos et al. (36)
PICP+PSA	PC	68	61	ELISA	78.00	96.00	0.970	Zissimopoulos et al. (36)
PICP	PC	42	6	RIA	54.00	93.00	0.840	Zissimopoulos et al. (37)
PSA	PC	42	6	RIA	68.00	91.00	0.880	Zissimopoulos et al. (37)
ICTP	BC	25	12	ELISA	56.00	93.00	/	Tähtelä et al. (38)
PICP	BC	25	12	ELISA	24.00	100.00	/	Tähtelä et al. (38)
PINP	BC	25	12	ELISA	30.00	98.00	/	Tähtelä et al. (38)
CEA	BC	164	200	ELISA+ TECAN	56.70	92.00	/	Wang et al. (39)
CA19-9	BC	164	200	ELISA+ TECAN	36.00	82.50	/	Wang et al. (39)
CA125	BC	164	200	ELISA+ TECAN	25.60	97.00	/	Wang et al. (39)
CA15-3	BC	164	200	ELISA+ TECAN	44.50	84.50	/	Wang et al. (39)
TPS	BC	164	200	ELISA+ TECAN	50.00	89.50	/	Wang et al. (39)
CEA+ CA19-9	BC	164	200	ELISA+ TECAN	67.10	78.00	/	Wang et al. (39)
CEA+ CA125	BC	164	200	ELISA+ TECAN	66.50	89.00	/	Wang et al. (39)
CEA+ CA15-3	BC	164	200	ELISA+ TECAN	68.90	88.00	/	Wang et al. (39)
CEA+ TPS	BC	164	200	ELISA+ TECAN	78.70	82.00	/	Wang et al. (39)
CA19-9+CA125	BC	164	200	ELISA+ TECAN	50.00	80.50	/	Wang et al. (39)
CA19-9+CA15-3	BC	164	200	ELISA+ TECAN	60.40	79.50	/	Wang et al. (39)
CA19-9+TPS	BC	164	200	ELISA+ TECAN	64.60	73.50	/	Wang et al. (39)
CA125+ CA15-3	BC	164	200	ELISA+ TECAN	52.40	91.50	/	Wang et al. (39)
CA125+ TPS	BC	164	200	ELISA+ TECAN	56.70	86.50	/	Wang et al. (39)
CA15-3+ TPS	BC	164	200	ELISA+ TECAN	63.40	85.00	/	Wang et al. (39)
Ferritin	NENpts	62	40	EIA	100.00	73.00	0.88	Rosiek et al. (40)
BMG	NENpts	62	40	EIA	100.00	46.00	0.74	Rosiek et al. (40)
CA125	NENpts	62	40	EIA	100.00	39.00	0.66	Rosiek et al. (40)
CEA	NENpts	62	40	EIA	50.00	98.00	0.70	Rosiek et al. (40)
AFP	NENpts	62	40	EIA	50.00	66.00	0.55	Rosiek et al. (40)
CA19-9	NENpts	62	40	EIA	67.00	59.00	0.52	Rosiek et al. (40)
CEA	lung cancer	133	562	Histopathology	76.77	86.33	0.67	Jiang et al. (41)
CA50	lung cancer	133	562	Histopathology	70.00	82.81	0.623	Jiang et al. (41)
CA125	lung cancer	133	562	Histopathology	87.72	72.97	0.748	Jiang et al. (41)
NSE	lung cancer	133	562	Histopathology	82.70	73.00	0.7	Jiang et al. (41)
Ferritin	lung cancer	133	562	Histopathology	92.20	75.40	0.619	Jiang et al. (41)
CYFRA21-1	lung cancer	133	562	Histopathology	54.70	73.70	0.697	Jiang et al. (41)
CEA	BC	54	49	qPCR	48.90	97.10	0.915	Mercatali et al. (42)
CA15-3	BC	54	49	qPCR	64.40	94.40	0.886	Mercatali et al. (42)
OPG	BC	54	49	qPCR	74.10	87.70	0.825	Mercatali et al. (42)
OPG+CEA	BC	54	49	qPCR	84.40	79.50	0.938	Mercatali et al. (42)
OPG+CA15-3	BC	54	49	qPCR	86.70	72.90	0.922	Mercatali et al. (42)
RANK-L	BC	54	49	qPCR	57.40	67.40	0.692	Mercatali et al. (42)
RANK-L+CEA	BC	54	49	qPCR	73.30	50.00	0.907	Mercatali et al. (42)
RANKL+CA15-3	BC	54	49	qPCR	75.6	47.20	0.894	Mercatali et al. (42)
RANK-L/OPG	BC	54	49	qPCR	40.70	77.50	0.70	Mercatali et al. (42)

### 2.1 AFP

AFP, known as hepatoembryonic antigen, is a biomarker for the identification of bone metastases (43). It plays a major role in embryonic and early embryonic development, but the adult owned the low level of AFP. AFP is commonly used as the diagnostic biomarker for liver, testicular, and ovarian carcinoma. Moreover, AFP can be used to predict bone metastases, which is a manifestation of antigen movement in a specific direction (43, 44). Studies showed that the serum level of AFP in patients with non-small cell lung cancer can be utilized to predict location-based tumor susceptibility and duration of location-based tumor treatment (45, 46). Another study showed that higher serum AFP level in the patients of cancer indicated the risk of bone metastases and thus to infer more effective cancer treatment options (44, 47). High level of serum AFP has been shown to help to diagnose patients with bone metastases with diagnostic accuracy of 75% as well as to predict tumor size, location, risk of metastases, and duration of treatment (40, 48). Recent studies have found that it can be utilized to assess location-based tumor susceptibility, as well as tumor size, location, and duration of treatment. To sum up, AFP is a significant biomarker for the detection of bone metastases.

### 2.2 CEA

CEA is a common antigenic factor that plays an important role in a variety of cancers, such as Colon cancer, stomach cancer, pancreatic cancer, small intestinal adenocarcinoma, lung cancer, liver cancer, breast cancer (49). CEA is a biomarker widely used in colorectal cancer screening and monitoring treatment response. However, its low sensitivity and specificity in bone tumors limit its application in bone metastasis. CEA is of particular importance in bone metastases. At present, CEA is used primarily to detect the occurrence and development of bone metastases, especially in breast cancer, lung cancer and gastrointestinal tumors (50, 51). CEA has excellent sensitivity and specificity, which can be used to assess the existence of bone metastases. The sensitivity and specificity of serum CEA were 19.0%-56.1% and 50%-92%, in the gastrointestinal tumors (39). At present, more and more studies have pointed out that CEA can help accurately diagnose bone metastases and improve the curative effect. Clinical trials have shown that increased CEA levels were linked to reduced efficacy in patients with breast cancer bone metastases (52, 53). In addition, CEA also has significant application value for clarifying tumor manifestations, namely the range of bone metastases and bone changes, so as to provide objective guidance for clinical treatment planning.

### 2.3 ALP and PSA

ALP and PSA are widely used to predict bone metastases of prostate cancer, but their accuracy and reliability in the diagnosis of bone metastases are inconsistent (54). Serum ALP is derived from osteoblasts with isoenzyme activities, which can hydrolyze phosphate esters. Moreover, serum ALP, can be used to indicate the specificity of reflecting bone metastases lesions, regarded as a biomarker of early differentiation of osteoblast precursor cells. ALP is specific biomarkers of bone tissue and widely utilized in bone tumors. The expression level of ALP can be used to estimate the balance between bone reconstruction and destruction. (55). Salter et al. found that ALP was oleophilic, which was an important biomarker reflecting osteoblast activity and tumor progression (56). Rao et al. suggested that ALP was a serum biomarker in predicting bone metastases of prostate cancer (57). Serum PSA, a serine protease, is commonly used in screening, diagnosis and efficacy evaluation of prostate cancer (58). In patients of prostate cancer with bone metastases, due to the proliferation of prostate cancer cells, a large amount of PSA was produced and secreted into the blood, resulting in elevated serum PSA (59, 60). PSA is a good indicator of bone metastases of prostate cancer. The higher the PSA, the greater the risk of bone metastases. When PSA < 20ng/ml, the risk of bone metastases was relatively small, while when PSA > 100ng/ml, the risk of bone metastases was higher than 80%. Therefore, further testing and prophylaxis were recommended when PSA > 20ng/ml (61). Although bone metastases are common sites of prostate cancer, the use of PSA in the diagnosis of bone metastases is limited.

### 2.4 CA and Rb

CA is used more frequently for the detection of breast and bowel cancer. CA199 is an important biomarker and apparent specific antigen for the detection of bone metastases of liver cancer. Studies have shown that the expression level of CA199 was related to the metastases of liver cancer, with the excellent ability to predict the malignant transformation and prognosis of liver cancer (39, 62). CA724 used for clinical diagnosis and prognosis of found guilty of an important tumor biomarker in breast cancer. Studies have shown that increased level of CA724 may represent increased bone metastases potential of breast cancer, which was more accurate for symptomatic radiotherapy (63, 64). CA50 is an apparent exclusive cancer biomarker used to detect bone metastases of liver cancer. The experimental results indicated that the level of CA50 can serve as a biomarker to predict the potential of bone metastases of liver cancer (65, 66). CA242 is an epigenetic specific antigen used to detect bone metastases in gastric cancer. Studies have indicated that increased level of CA242 can be used to predict bone metastases in gastric cancer, and can effectively help to improve the treatment efficiency and anti-cancer therapeutic effect of tumors (67, 68).

Rb is widely used in the diagnosis of bone-derived tumors, whose reduced expression indicates an increased risk of bone metastasis. (69). P53 is a tumor suppressor gene protein that is abnormally expressed in a variety of tumors. NM23 is an RNA-binding protein that is abnormally expressed in non-small cell lung cancer and some other cancers, whose application in bone tumors is restricted.

In conclusion, the current researches on protein biomarkers of bone metastases are still in the primary stage. Despite the fact that some biomarkers have been proved to have certain application value, more biomarkers need to be explored and applied in the accurate diagnosis of bone metastases and the formulation of treatment plans.

## 3 Application of ncRNA as biomarkers in bone metastases

With the development of high-throughput sequencing technology and bioinformatics, a large number of ncRNA, such as miRNA, lncRNA and circRNA, have been found to be involved in gene expression regulation, cell differentiation, etc (70, 71). In addition, they are closely related to the occurrence and development of tumors.

### 3.1 miRNA

miRNA in mammalian serum and plasma have high stability and can be stable under repeated freeze-thaw and different pH conditions (70, 72–74).

miRNA plays an important role in the diagnosis of bone metastases, which can help doctors to identify cancer metastases to bone in order to provide timely treatment (70, 71). Currently, many studies have shown that the expression level of miRNA from samples can be used to identify the presence of bone partially implanted cancer cells (70, 72, 75–77). Some miRNA such as let-7 (78, 79), miR-125b (80, 81), and miR-21 were significantly expressed in experimental tumor migration into the mouse bone, contributing to the identification and diagnosis of bone metastatic cancer (82–84). miRNA plays an important role in tumor therapy, and it has attracted more and more attention as new therapeutic biomarkers (85, 86). Targeting miRNA therapy can reduce drug toxicity and achieve higher efficacy by accurately identifying and treating bone metastases. Contemporary studies have shown that miRNAs-based therapy has a significant promoting effect on inhibiting the growth, invasion and immune resistance of bone metastases (7, 87). Currently, miRNAs that have been considered as biomarkers of bone metastases include miR-21, miR-141, miR-221/222, miR-24, miR-20a, miR-145, miR-29a, miR-26a, miR-22, miR-125b, miR-15b, miR-193b, miR-196a, and miR-101 et al., which were shown in **Table 3**.

**Table 3:** Application of ncRNA biomarkers of bone metastases.

Biobiomarker	Bone transition stage	Primary cancer types	Study population characteristics		Clinical application	Deregulation	Ref.
			Cases group	Controls group			
miR-192-5p	Mixed	LC	68	78	Early diagnosis and prediction of bone metastases.	Low-expression	Zou P et al. (88)
miR-335	Osteoblastic	SCLC	10	5	Diagnosis of bone metastases in prostate cancer, miR-335 might target cytokines linked to osteoclast induction and bone turnover.	Over-expression	Gong et al. (89)
miR-139-5p	Osteoblastic	NSCLC	25	30	As a biobiomarker and treatment target in monitoring and controlling bone metastases.	Down-regulated	Xu et al. (90)
miR-139-5p	Mixed	EWS	19	/	Down-regulation of miR-139-5p is associated with disease progression in EWS and may serve as a risk assessment biobiomarker.	Down-regulated	Roberto et al. (91)
miR-124-3p	Mixed	EWS	19	/	Down-regulation of miR-124-3p is associated with disease progression in EWS and may serve as a risk assessment biobiomarker.	Down-regulated	Roberto et al. (91)
miR-584-5p	Mixed	EWS	19	/	Down-regulation of miR-584-5p is associated with disease progression in EWS and may serve as a riskassessment biobiomarker.	Down-regulated	Roberto et al. (91)
miR-7	Osteolytic	BC	51	4	Promoting cancer cell progress and consequently results in NSCLC growth. miR-7 may become promising molecular therapies in NSCLC treatment.	Down-regulated	Vimalraj et al. (92)
let-7c	Mixed	LAC	/	/	Low levels of let-7c expression and metastases, venous invasion, advanced TNM stages and poor survival of NSCLC patients.	Down-regulated	Zhao et al. (79)
miR-10b	Osteolytic	BC	122	59	An independent prognostic factor in NSCLC patients.	Up-regulated	Zhao et al. (93)
miR-17 family	Osteolytic	OS	75	/	Not only decrease cisplatin-resistant but also reduce migration by inhibiting EMT in A549/DDP cells.	Over-expression	Arabi et al. (94)
miR-21	Osteolytic	OS	65	/	Regulate the biological characteristics of tumor cells and the ability of bone metastases.	Low-expression	Yuan et al.(82)
miR-16/miR-15a	Osteoblastic	PC	99	5	miR-15/miR-16 control organ-confined and distant invasion of prostate cancer cells.	Over-expression	Bonci et al.(83)
miR-141	Osteoblastic	PC	52	89	Inhibit the growth of osteoclasts by inhibiting the synthesis of bone morph regulatory factors.	Down-expression	Huang et al. (69)
miR-221/222	Mixed	PC	18	3	Actively involved in bone metastases of cancers such as prostate cancer and breast cancer.	Low-expression	Xu et al. (95)
miR-24	Osteolytic	OS	/	/	Affect the onset, development and subsequent therapeutic effect of bone metastases.	Over-expression	Liu et al. (2017) (96)
miR-20a	Osteolytic	OS	10	8	Enhance immune function, reduce inflammatory response and promote the body’s immune response to tumors.	Over-expression	Koshkina et al. (97)
miR-145	Osteoblastic	ESCC	19	19	Affecting the migration and reproduction of cancer cells in bone marrow, and helping to inhibit the occurrence of bone metastatic tumors.	Over-expression	Cui et al. (98)
miR-29a	Osteoblastic	SCLC	10	/	Inhibit the mechanism of cancer cells, and inhibit the migration and reproduction of cancer cells in bone marrow, thus inhibiting the occurrence of bone metastatic tumors.	Over-expression	Gong et al. (89)
HOTAIR	Mix	BC	/	/	HOTAIR affects and blocks the growth, metastasis and apoptosis of breast cancer cells through the miR-20a-5p/HMGA2 axis	Down-expression	Zhao et al. (99)
circITGA7	Mix	OS	/	/	circITGA7 may be involved in the occurrence and development of bone metastases	Down-expression	Fang et al. (100)

#### 3.1.1 miR-21

miR-21 has been extensively studied as a key biomarker for various types of cancer, including breast, lung, prostate, ovarian, and colorectal cancers (82–84). One study found that miR-21 was significantly up regulated in bone metastases tissue samples, compared to primary tumor tissue samples from patients with breast cancer (9). Furthermore, they observed that serum levels of miR-21 were significantly higher in breast cancer patients with bone metastases. They suggested that miR-21 could be used as a non-invasive biomarker to detect bone metastases in breast cancer patients. Similarly, another study found that miR-21 was over expressed in bone metastases tissue samples from patients with prostate cancer. They observed that miR-21 expression was positively correlated with bone metastases, suggesting that miR-21 could be used as a prognostic biomarker to predict the progression of bone metastases in prostate cancer patients (101). One study analyzed miR-21 expression in serum samples from patients with breast cancer and bone metastases, as well as healthy controls, drawing a conclusion that serum levels of miR-21 were significantly higher in breast cancer patients with bone metastases, compared to healthy controls. (102). Overall, the above studies suggested that miR-21 was a promising biomarker in the detecting and monitoring of bone metastases in various types of cancer. Its potential use as a therapeutic target warrants further investigation in preclinical and clinical studies.

#### 3.1.2 miR-141

miR-141 has been a top priority in the study of bone metastases in recent years (103, 104). miR-141 can inhibit adenovirus transcription factors, immune response and apoptosis-mediated response, and exert a huge effect on inhibiting tumor growth to promote factor expression and inhibit gene expression regulation (105, 106). Studies have shown that miR-141 is paramount in preventing the development of bone metastases (106). In previous studies, miR-141 can prohibit the growth of osteoclasts by inhibiting the synthesis of bone morph regulatory factors, thus delaying the metastases process (107, 108). Meanwhile, miR-141 interdicted the migration and invasion of bone metastases. In addition, miR-141 can also induce tumor cell apoptosis, thus playing a momentous role in the process of bone metastases (109, 110). In conclusion, miR-141 is instrumental in inhibiting the development of bone metastatic tumors and may be essential in clinical diagnosis and treatment of bone metastatic tumors in the future.

### 3.2 lncRNA and circRNA

lncRNA and circRNA are a class of emerging ncRNA, playing important roles in the occurrence and development of human diseases. In recent years, more and more studies have shown that lncRNA and circRNA may also be strong candidates for tumor biomarkers of bone metastasis. There are some studies have found that lncRNA is crucical in bone metastasis. For example, one research has shown that metastasis-associated lung adenocarcinoma transcript 1 (MALAT1) can promote tumor cell invasion and migration, whose expression level was elevated in patients with bone metastasis (111). Other lncRNAs such as HOX antigens intergenic RNA (HOTAIR) and taurine unregulated gene 1 (TUG1) have also been found to be closely associated with the occurrence and development of bone metastases. HOTAIR affected and blocked the growth, metastasis, and apoptosis of breast cancer cells through the miR-20a-5p/HMGA2 axis. In the past few years, studies have found that lncRNA-SOX2OT may have clinical diagnostic value and can be employed as an *in vitro* diagnostic biomarker for bone metastases (110). It was found that the level of lncRNA-SOX2OT in serum in patients with bone metastases were significantly higher than those in the control group (112). Besides, studies had found that lncRNA-SOX2OT might regulate the phenotype of bone metastatic tumor cells. It was also found that lncRNA-SOX2OT inhibited the expression of MMP-13, which explained why lncRNA -Sox2OT may be associated with the regulation of bone metastases (113). Moreover, by combining multiple gene factors, we found that HIF-1, Hypoxia, and LCC-Sox2OT gene regulatory networks may present in bone metastases. What’ more, the researchers suggested that the expression of LCC-Sox2OT may be related to cell status, which can be used to identify biomarkers *in vitro*, and to identify and forecast the incidence of bone metastatic tumors *in vivo* (54, 112, 114, 115).

In contrast, circRNA has been relatively poorly studied in bone metastasis (99). What’s more, some studies have shown that circRNA may also be a biomarker of bone metastases. For instance, there reported a study showing that circITGA7 (circular RNA-integrin subunit alpha 7) may be involved in the occurrence and development of bone metastases. This circular transcription can inhibit apoptosis of a variety of cells, whose expression level was significantly increased in patients with bone metastasis (100). Of course, studies on tumor biomarkers for bone metastases in lncRNA and circRNA are still in the preliminary stage, and their potential mechanisms and clinical application value need to be further verified and explored.

## 4 Bone metastasis biomarkers in liquid biopsy

Compared with traditional tissue sample biopsies, liquid biopsy-based markers have the following advantages:1. Non-invasive: Liquid sample collection is relatively simple, such as blood, urine, etc., without tissue excision or puncture, which can reduce patients’ pain and risk. 2. Systemic: Liquid samples can reflect the situation of the whole body, avoiding local errors in the collection of tissue samples, making them more representative and comprehensive. 3. High sensitivity: the concentration of markers in liquid samples is relatively stable and is not affected by tissue heterogeneity, making the detection results more accurate and reliable. 4. Good repeatability: liquid sample collection is relatively simple and non-invasive, which can be collected multiple times to monitor tumor growth and metastasis. 5. Forward-looking: in the detection and monitoring of early tumors, liquid biopsy can provide a more flexible and sensitive detection method, and improve the rate of early diagnosis and treatment of tumors. For tumor biomarkers of bone metastasis in liquid biopsy, molecular indicators related to bone metastasis, such as ctDNA, exosomes and circulating tumor cells (CTCs), were mainly screened from biological fluids such as blood or urine. These indicators have the advantages of high sensitivity, non-trauma and dynamic monitoring, which can be utilized to achieve early detection, monitor and prediction of bone metastasis. Corresponding contents were shown in **Table 4**.

**Table 4:** Bone metastasis tumor biomarkers in liquid biopsy.

Biobiomarker	Bone transition stage	Primary cancer types	Clinical application	Deregulation	Ref.
CD44	Osteolytic	SCLC	An important role as an early diagnostic biomarker and prognostic indicator of bone metastases.	Over-expression	Zhao et al. (116)
CXCR4	Mixed	LC	Associated with metastases of tumor cells to bone tissue and can be used as an essential biomarker of bone metastatic tumors.	Over-expression	Chai et al. (54)
CD74	Osteoblastic	NSCLC	Predict the pathological changes of tumors and the prognosis of tumor patients after treatment.	Up-regulated	Loreth et al. (2021) (117)
Mesothelin /CK19	Osteoblastic	ESCC	Diagnose and predict the development of tumors.	Over-expression	Zhang et al. (2010) (118)
Osteopontin /CAIX	Osteoblastic	BC	Assess the risk of tumor invasiveness and metastases.	Low-expression	Jiwa et al. (2014) (119)
CXCR4	Mixed	Gastrointestinal malignancies	Associated with metastases of tumor cells to bone tissue and can be used as an essential biomarker of bone metastatic tumors.	Over-expression	Roberto et al. (91)

### 4.1 ctDNA

ctDNA is a piece of DNA which was released into the blood by cancer cells with certain specificity and sensitivity. ctDNA is a piece of DNA that is released into the bloodstream when cancer cells die or die. Unlike normal plasma DNA, ctDNA contains specific variations from tumor cells. Therefore, ctDNA can be used as a non-invasive “liquid biopsy” method, which can be widely used in the early diagnosis, treatment monitoring and prognosis assessment of tumors. ctDNA has the following advantages: 1. Non-invasive: ctDNA sampling is simple and non-invasive, requiring no painful tissue removal or cancer cell culture. 2. High sensitivity: The proportion of ctDNA in the blood is very low, so it can be detected even in the mild disease, especially in the primary tumor detection has a better application prospect. 3. High specificity: ctDNA contains specific variations from tumor cells, which can distinguish different subtypes and tumors at different stages of synchronization. 4. Real-time dynamic monitoring can be realized: ctDNA can reflect real-time treatment progress, drug resistance and relapse, which can provide doctors with better treatment strategies. To sum up, ctDNA as a tumor marker has great advantages and has gradually become a hot spot in cancer research.

In the detection of bone metastases, studies on ctDNA as a kind of biomarker in bone metastases mainly focus on the following aspects. ctDNA tests based on gene mutations. Firstly, some mutations associated with bone metastases, such as the fatty acid acylase gene (ACSL5) and the fusion gene TMPRSS2-ERG, had been shown to have high sensitivity and specificity when ctDNA was detected in the blood. These mutations were valuable for the detection of bone metastases (120). For example, one study found that ctDNA, which detected a deletion of the *PTEN* and mutation of the *TP53*, had high sensitivity and specificity in the plasma of prostate cancer patients. Secondly, the detection of ctDNA is based on epigenetic changes. Bone metastasis is also closely associated with epigenetic changes in DNA methylation and histone modification. Studies had shown that some epigenetic biomarkers such as *RASSF1A* (121), *IGFBP-3* (74), *MGMT* and ctDNA of *GSTP1* can be detected in patients with bone metastases. These biomarkers provided an accurate value for the early detection and evaluation of bone metastases. Finally, the detection of ctDNA based on microsatellite instability (MSI), which is usually caused by the depletion of mismatch repair systems *in vivo* and is a hallmark of many familial non-multiple systemic tumors. It has been noted that the appearance of MSI in cancer cells is closely related to the occurrence and development of bone metastasis. There was a study showed that the detection of MSI in ctDNA could be used to evaluate the prognosis of bone metastases in intestinal cancer, providing a reference for the selection of treatment (122). In conclusion, the research and application of ctDNA as tumor biomarkers in bone metastases are developing and improving all the time. Although it still faces some technical and methodological bottlenecks, future studies will continuously improve its application prospect and clinical value. It is expected to become an important indicator in the timely detection, prognosis assessment and treatment monitoring of bone metastases.

### 4.2 CTCs

CTCs are cells shed from tumors and enter the peripheral blood of the body, which are the highest manifestation of the spread of malignant tumors. The genetic characteristics or antigens of CTCs are identical to those of primary tumor cells, but the method of obtaining CTCS is less invasive and highly reproducible (123). Systematic monitoring of CTCS through liquid biopsies enables monitoring of disease processes, detecting emerging resistance genes, and identifying new molecular targets (124). Relevant studies had shown that CTCs were highly invasive and malignant, and could evade immune surveillance of the body. CTCs can reflect the characteristics of tumor metastases and disease changes in patients with malignant tumors, playing crucial part in the curative effect and recurrence prediction of malignant tumors, so as to provide a reference for the early diagnosis and treatment of diseases (125). Detection of CTCs is a prerequisite for distant metastases of solid tumors (126). The specific contents were shown in **Table 4**.

Taking CD44 for example. CD44 is a protein, which is deemed to be a pathological indicator. It is generically known as CD44 receptor, also known as adhesion molecule, which is a variety of tumor cell adhesion molecule genes, associated with signal activation and cell cross-coupling of cell molecules (127, 128). Clinical studies had shown that CD44 was a diagnostic biomarker and prognostic indicator in a variety of tumors, including liver cancer, stomach cancer, esophageal cancer, ovarian cancer, prostate cancer, etc. It can be found in blood, cellular mediators, tissue biopsy specimens, tumor cells, and normal cells (129, 130). Studies had shown that the expression of CD44 was related to the expression of late genes such as *PD-L1*. Its expression may also matter in the early detection of tumors and later forms of metastases. Laboratory studies have demonstrated that CD44 can form binding with chemical factors of mitogen and cell surface, improve cell binding to other cell surface molecules and thus increase the risk of bone metastases (127, 131).

### 4.3 Exosomes

Extracellular vehicles (EVs) include apoptotic bodies (ABs), microvesicles (MVs), and exosomes, encapsulate tumor-specific content, and transmit them into environmental cells and circulation. Exosomes as molecular biomarkers, play major roles in diagnostic decisions and treatment selection in the detection of cancer bone metastases (132). Exosomes have relatively stable components that confer biological effects on adjacent or distal cells. Exosomes are also nanoparticles secreted by all cell types (133, 134). Due to their nature as nanovesicles, exosomes can be transferred proximal and distal across different biological barriers. Exosomes have been used as transport carriers for a variety of molecules including proteins and different RNA (135).

Therefore, exosomes can be used not only as reaction markers of different diseases and physiological states, but also as tools of *in vitro* genetic engineering for the treatment of different diseases and organs. This shows that exosomes, as communication mediators between cells, have infinite potential as biomarkers. From the perspective of exosome functioned as molecular biomarkers, exosomes function importantly in the molecular linkage of bone metastases tumor, accurate detection and quantification of bone metastases tumor biomarkers, which are extremely important (136). On the one hand, the studies of exosome molecular biomarker will provide useful information that can help clinicians more accurately in diagnosing bone metastases. Exosomes can be detected diagnostic cancer biomarkers in body fluids, such as prostate specific nucleic acid expression (PNA), gastrointestinal specific protein expression (GIP), and respiratory specific nucleic acid expression (RNA) (137, 138), which can identify cancer cells faster and more accurately, providing more detailed and reliable molecular information of cancer cells, so as to better predict the trend of cancer cell metastases and provide more accurate treatment guidance.

miR-375 and miR-141, which from exosomes, are the main biomarkers of bone metastases, which are mainly involved in regulating the respiration and proliferation of cancer cells (7, 57). The increased expression of miR-375 can promote the malignant proliferation of cancer cells. On the contrary, miR-141 will promote and inhibit the proliferation of cancer cells, reduce the damage to sensitive cancer cells, and decrease the resistance to drug-resistant cancer cells (139). In addition, TM256, LAMTOR1 and VATL were tumor biomarkers associated with miR-141 and miR-375. TM256 can recognize the increased expression of miR-141 and promote the proliferation and growth of cancer cells (103). LAMTOR1 can recognize the increased expression of miR-141 and miR-375 and inhibit the proliferation and growth of cancer cells (69). VATL can recognize the increased expression of miR-375 and promote malignant proliferation of cancer cells. ADIRF was a specific tumor biomarker that can detect and recognize increased expression of miR-375 and miR-141, thereby contributing to the growth and proliferation of cancer cells (104, 140).

## 5 Application of other kinds of biomarkers in bone metastases

DNA methylation is a joint biological modification that affects gene expression by introducing methyl groups into DNA molecules through methylase. In tumor cells, the change of DNA methylation degree is closely linked to tumor growth, cell proliferation and development. Currently, there are many biomarkers of bone metastases based on DNA methylation, which include many different types. Glutathione S transferase P1 (GSTP1) is an antioxidant enzyme whose DNA methylation leaded to decreased expression levels, which had been demonstrated in many tumor cases, including bone metastases (141). SEPT9 was often considered a biomarker of DNA methylation. Recent studies had shown that exon 8 methylation of SEPT9 was a valid biomarker for blood samples (both venous and serum) from lung cancer patients (141, 142). The HOXB gene family is a member of the HOX gene superfamily, and HOXB7 may acted as a proto-oncogene in a variety of malignancies (143). DNA methylation of HOXB7 gene played an essential role in bone metastasis of prostate cancer cells (144). That is to say, DNA methylation of bone metastases tumor biomarkers provides a novel idea and means for the diagnosis, monitor and treatment of bone metastases. However, more studies are required to confirm their clinical application prospects as well as their sensitivity, specificity and stability.

Histone methylation is a key epigenetic modification, which plays a balancing and regulating role in gene transcription and expression. Tumor markers of bone metastases targeted at histone methylation mainly include the following aspects. H3K9me3 is the triumphalist form of the 9th lysine of histone H3 and is a silencing marker for many genes. The loss or reduction of H3K9me3 in bone metastases may be related to its enhanced ability to metastasize and the difference in prognosis (145). H3K27me3 is the triumphalist form of the 27th lysine of histone H3, which plays an important role in cell growth and differentiation. Reduction of H3K27me3 in bone metastasis may lead to inhibition of apoptosis and the growth and metastasis of cancer cells (146). H3K4me3 is the triumphalist form of lysine at the fourth position of histone H3, which is a marker of enrichment in genes with high transcriptional activity. During the treatment of patients with bone metastases, prominent expression of H3K4me3 was associated with the prognosis and progression of bone metastases (147). In conclusion, the study of histone methylation tumor markers of bone metastasis provides a novel idea and means for the early detection and treatment of bone metastasis. Although there are still some challenges in the application, they are expected to be one of the principal markers of bone metastasis in the future.

## 6 Perspectives and future opportunities

This paper mainly introduces the commonly used clinical protein biomarkers, ncRNA, and liquid biopsy biomarkers. Each type has its specific advantages, limitations in the clinical application. Protein-based tumor biomarkers have been extensively studied and have a wide range of applications, including diagnosis, disease surveillance and therapeutic strategies. Numerous protein measurement techniques and automated methods have been rapidly developed, making high-throughput identification and measurement easy and fast. Proteins can be interfered with by external factors (such as diet and preparations), and in some cases of proteins may be non-specific, which can lead to false positives. So, the interpretation of the results does not necessarily reflect accurate. Compared with proteins, the structure and function of ncRNAs are still being studied, so understanding the role of ncRNAs and their detection techniques are limited. Some ncRNAs may be raised at similar levels in multiple tumor types and non-tumor diseases, so there may be some limitations in the differential diagnosis process. To sum up, these types of biomarkers have their peculiar advantages and disadvantages, and the future development will be different depending on the specific application. Among them, miRNA, as an emerging method, may be the future direction while further understanding its biological role and mechanism. Due to the wide variety of biomarkers, this study mainly elaborated protein, ncRNAs, liquid biopsy biomarkers and other studied biomarkers, which were mainly derived from serum plasma and tissue. Our team will conduct a more comprehensive and detailed description of such biomarkers in subsequent studies, so as to provide reference for the clinical application of biomarkers of bone metastases and the early diagnosis of diseases.

Future research on how to find new methods of screening and detecting biomarkers, and the set of cut-off value, etc., not only for detection but also for prognosis is needed. Firstly, large-scale prospective clinical studies are required. More large-scale prospective clinical studies are needed to confirm the sensitivity, specificity, and stability of different markers, as well as their feasibility for early detection, classification, and treatment of bone metastases. Secondly, combinations of multiple biomarkers can be studied. Combined with biomarkers of different types of bone metastases, a more accurate diagnosis and prediction model was established. In the process of integration, it is necessary to investigate the interaction, influence and cooperation among different biomarkers, and establish the corresponding bioinformatics model and algorithm combined with bioinformatics. Finally, multidisciplinary cooperation and communication is important. There is necessary to have closer collaboration among clinicians, basic scientists, bioinformatics specialists and engineers to leverage their expertise and skills to better support the research and application of markers for bone metastases.

In conclusion, in the future, the study of bone metastases tumor markers will gradually develop from a single biomarker study to a systematic and integrated research model, so as to more accurately and comprehensively understand the biological characteristics and clinical manifestations of bone metastases, promoting more significant progress in the diagnosis and treatment of bone metastases.

## Author contributions

JL and HL conceived the research. YH and FZ conducted the study and drafted the manuscript, and they contributed equally to this work. YM, YL, YZ, NY, ML contributed to the acquisition, or interpretation of data and critically reviewed and revised the article for important intellectual content. All authors contributed to the article and approved the submitted version.
